# Proposed medical school curricula for 3D printing

**DOI:** 10.1186/s41205-025-00306-6

**Published:** 2025-12-02

**Authors:** Danielle Heiser, Stacy Ruther, Owais Salahudeen, Taaha Adamji, Jordan Mackner, Liane Ruddy, Garrett Trang, Prashanth Ravi, Jonathan M. Morris, Summer J. Decker, Jonathan M. Ford, David H. Ballard, Kimberly Hatch, Michael F. Morris, Richard L. Hallett, Frank J. Rybicki, Rayna DeBellevue

**Affiliations:** 1 Department of Radiology, University of Arizona College of Medicine – Phoenix, Phoenix, AZ USA; 2 Department of Radiology, University of Cincinnati College of Medicine, Cincinnati, OH USA; 3 Department of Radiology, Mayo Clinic Alix School of Medicine, Rochester, MN USA; 4 Department of Radiology, Keck School of Medicine of USC, Los Angeles, CA USA; 5 Mallinckrodt Institute of Radiology, Washington University School of Medicine, St. Louis, MO USA; 6 Banner University Medical Center – Phoenix, Phoenix, AZ USA

**Keywords:** 3D printing, Medical education, Anatomy, Curriculum development, Medical training

## Abstract

**Background:**

3D printing is increasingly utilized in medical education, providing a hands-on approach to anatomical learning, surgical planning, and interdisciplinary collaboration. Despite growing interest, standardized curricula incorporating 3D printing into medical education are lacking.

**Methods:**

A multidisciplinary team of medical educators, engineers, and students developed three distinct curricular models to integrate 3D printing into undergraduate medical education. These models include (1) integration into anatomy coursework, (2) a fourth-year clerkship elective, and (3) a pre-clerkship elective. The outline for each curriculum was designed to be adaptable across institutions, emphasizing hands-on learning, imaging segmentation, basic elements of computer-aided design (CAD), and 3D printing applications in clinical care.

**Results:**

The proposed curricula outlined provide structured pathways for incorporating 3D printing into medical education, enhancing student engagement and comprehension of complex anatomical structures. By integrating 3D printing into anatomy courses, clerkships, and elective rotations, students gain critical skills applicable to future clinical practice. The curricular models vary in scope and resource requirements, offering flexibility for adoption across medical schools.

**Conclusions:**

Standardizing 3D printing curricula in medical education enhances anatomical understanding, promote interdisciplinary collaboration, and prepare students for future applications of this technology in clinical practice. Our framework serves as a guide for institutions seeking to implement 3D printing curricula, fostering innovation and hands-on learning opportunities for medical trainees.

**Trial registration:**

Not applicable.

**Clinical trial number:**

Not applicable.

**Supplementary Information:**

The online version contains supplementary material available at 10.1186/s41205-025-00306-6.

## Introduction

3D printing is increasingly used for medical education [1]. A medical ‘makerspace’ is common in medical schools, either integrated into a digital medical library, as part of a larger laboratory that includes virtual and augmented reality, or as a stand-alone printing facility. Medical students not only express a strong interest in learning 3D printing but also recognize its value in enhancing their medical education and career development. Medical students are eager to generate and use surface mesh files, either to enhance their knowledge, to be competitive in post-graduate applications, or to learn about subspecialty medical devices (e.g., surgical cutting guides). With this interest and utilization of surface mesh files and 3D printing, there is an unmet need to propose, standardize, and implement one or more curricula for medical students.

A multidisciplinary team of medical school and hospital leaders, engineers, subspecialty providers, and medical students proposed three curricula that can be translated to medical education programs and modified, as needed. The curricula proposed are intended to be complemented by reading material from the most widely downloaded and recognized textbook in the field [2]. Makerspaces are common in many educational portfolios, providing 3D printing resources for a variety of students at very low (or no) cost. Examples of these modifications can be education for a residency program in radiology, general surgery, oral and maxillofacial surgery, or orthopedics. Additionally, clinical fellows (cardiology, interventional radiology) participate in patent-specific 3D printing routinely used for planning and treating complex patients.

A medical makerspace can be created at a medical school with modest resources, namely one engineer and one provider medical champion. Of note, the medical champion can be “learning” the material and techniques during the development of the lab if the medical school does not have a medical champion at the time the program is launched. This project outlines the proposed medical curriculum; the details of implementation will be developed organically based on the preferences of individual institutions.

This project also includes recommendations for physical space and its requirements as well as the hardware, software, and consumables needed to launch the makerspace. Both material extrusion and inverted vat polymerization are included in the physical space; only “desktop” 3D printing is suggested at the outset. This lowers the physical footprint and budget. A smaller project can launch with only material extrusion. Invariably, the 3D printing program will expand and mature, and with that growth will come additional technologies. In addition, desktop 3D printing is likely to be supplemented with larger hardware to accommodate larger parts and to add complexity such as multiple color and multiple material printing.

### Clinical example: maxillofacial reconstruction

**Fig. 1:**
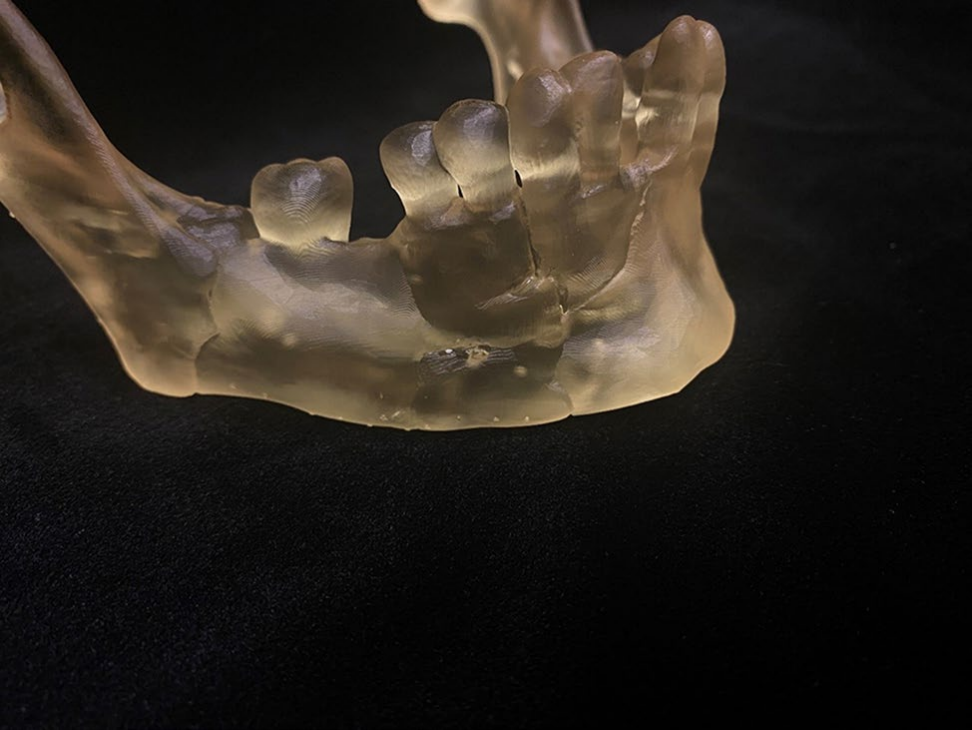
3D printed premorbid mandible used in preparation of craniomaxillofacial surgery

**Fig. 2:**
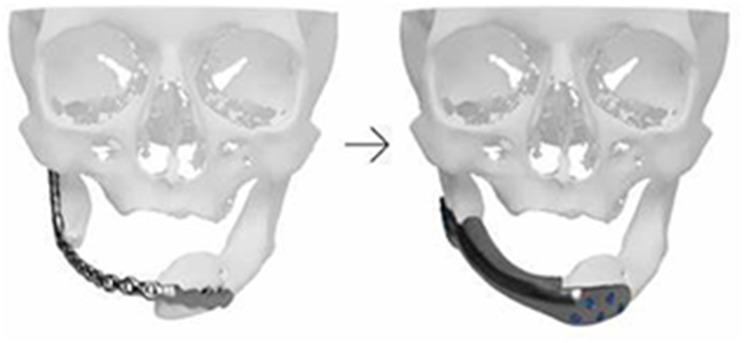
3D printed model used to pre-bend fixation plates for surgical reconstruction

While radiology plays a critical role in the preoperative planning and management of complex maxillofacial trauma, 3D printing provides an opportunity for hands-on medical student engagement. A well-established example of a student project focuses on mandibular reconstruction. In this case, volumetric CT imaging with thin-slice (e.g. 1 mm) reconstructed images of a patient with a complex mandibular fracture is used to generate volumetric data for segmentation. The medical students participate in the image processing workflow, learn to isolate fractured bone fragments, and subsequently digitally reconstruct the mandible to its premorbid state (Fig. 1). The resulting surface mesh files are printable. After fabrication, the 3D-printed model is used to enhance anatomical visualization, aid in preoperative planning, and guide the pre-bending of fixation plates for surgical reconstruction (Fig. 2). Through involvement in segmentation, model creation, and clinical application, students gain direct experience in radiology-driven surgical planning while developing technical skills in 3D printing and image post-processing. This example shows how medical students can actively engage in radiology-based projects, bridging the gap between diagnostic imaging, procedural planning, and hands-on medical education.

## Methods

Before developing the following curricula, a group of third- and fourth-year medical students formed a student-led interest group. The ‘home’ medical school (University of Arizona College of Medicine– Phoenix, Phoenix, AZ, USA) facilitates student assembled and student interest groups, including one for radiology and the new group focused on Digital Health and 3D printing. Interest groups have faculty mentors, through which collaboration was established from other leading medical centers.

Students desire to both learn about 3D printing and improve medical education. As individuals who will undoubtedly encounter and utilize this technology in their future careers, they are highly motivated to create an educational plan that can be widely used to educate future physicians on how surface mesh files, digital health, and 3D printing enhance personal learning and their ability to care for future patients.

With local mentorship supplemented by mentors nationally, students become familiar with the 3D printing process from start to finish, along with some of its many applications. This led to brainstorming where this learning could fit into the local medical education. This was expanded to the proposed curricula that can be adopted and modified locally.

The student group identified opportunities that complement existing curricular components at the ‘home’ medical school: the Clinical Anatomy course, a fourth-year clerkship elective, and a pre-clerkship elective block that takes place at the end of the first year. Students also gathered an understanding of how 3D printing has been incorporated in medical education elsewhere. While most commonly this appeared in residency training, the literature notes that the following medical schools do incorporate 3D printing into their undergraduate medical education: Mayo Clinic Alix School of Medicine [3], University of Michigan Medical School [4], and University of Illinois College of Medicine [5]. As these programs demonstrate, effective 3D printing education requires more than just access to printers—it must incorporate hands-on learning at every stage of the process, including segmentation, basic elements of computer-aided design (CAD), 3D printing, and post-processing the physical parts. By engaging with each step, students gain a comprehensive understanding of how medical imaging data is transformed into surface mesh files that can then be 3D printed to create physical models for educational and clinical applications.

Using these schools as an example, students further brainstormed how they could adapt these ideas into an approach that can be applied more universally. This culminated in the following proposed curricula.

## Results

There are three proposed curricula (Table 1). Each can be broadly applied to various undergraduate medical education programs. They provide foundational skills in emerging technology, enhanced visualization and understanding of challenging medical subjects, opportunities for interdisciplinary learning, and an additional avenue for active learning and engagement.

The specific structure of educational programs will vary across institutions, as each medical school should develop an approach that aligns with its delivery of educational materials as well as its assessment (and as applicable, grading) philosophy. For instance, some medical schools do not have formal “graded” evaluations for electives, whereas others have required assignments and grades. Implementation should also be tailored to institutional preferences regarding instructional format (in-person versus virtual or pre-recorded, traditional versus flipped classroom approach), evaluation and assessment methods, faculty feedback, and deliverables such as printed models, presentations, and/or written reflections.

Incorporating new technology into medical education has challenges. Integration of any of these options will entail additional resource requirements in the form of hardware, software, materials, personnel, and space, which ultimately limits scalability and accessibility.

**Table 1:** Overview & elements of 3D printing curricula

Curricular Options	Primary Outcome	Learning Objectives	Schedule	Strengths	Limitations
**Integration into Anatomy**	Use of medical imaging & 3D models to learn anatomy	- Understand anatomical structures through 3D models - Enhance anatomical learning through hands-on activities - Acquire technical skills in 3D printing	Session 1: Cardiovascular Session 2: Abdomen & Pelvis Session 3: Musculoskeletal Session 4: Head & Cranial Nerves	- Provides comprehensive knowledge base of 3D printing fundamentals - Reaches all medical students - General consensus that this is the ideal approach	- Most resource intensive - Requires time allocated to multiple faculty members - Changing existing anatomy curriculum is “biggest lift,” starting with electives can pave the road
**4th Year Clerkship Elective**	Advanced application of 3D printing to clinical care	- Apply 3D printing for clinical decision-making - Collaborate effectively in multidisciplinary teams - Develop project management skills - Assess how 3D printing is used to fabricate medical devices used in future career	Week 1: Introduction & Basic Printing Week 2: Anatomical & Procedural Applications Week 3: Hands-on Printing & Clinical Integration Week 4: Project Development & Presentation	- Pilot for highly engaged students - Students get more direct attention from faculty	- Smallest cohort for same initial resource investment
**Pre-Clerkship Elective**	Learn how to convert an image into a 3D printed part	- Master 3D printing technology basics from design to printing - Develop proficiency in converting CT and MRI to printable file types - Enhance learning through 3D models	Week 1: Introduction to 3D Printing Week 2: Anatomical & Surgical Applications Week 3: Hands-on Printing	- Earlier student exposure - Potentially more students get exposure overall (can have more cohorts in an academic year given shorter duration)	- Less total curricular hours - Limited cohort size - Faculty less engaged with student projects due to shorter duration

## Discussion

3D printing is valuable for medical education. The Liaison Committee on Medical Education (LCME) accredits US medical education, mandating content on modern biomedical sciences and organ systems [6]; 3D printing aligns with LCME standards by supporting anatomy and organ system learning.

Medical students desire a makerspace as a learning center, they desire a resource for creativity, and finally they desire a means to promote scholarship. These facilities are proven to enable trainees to gain experience in a subspeciality or to gain a strategic advantage in post-graduate applications [7–10].

**Fig. 3:**
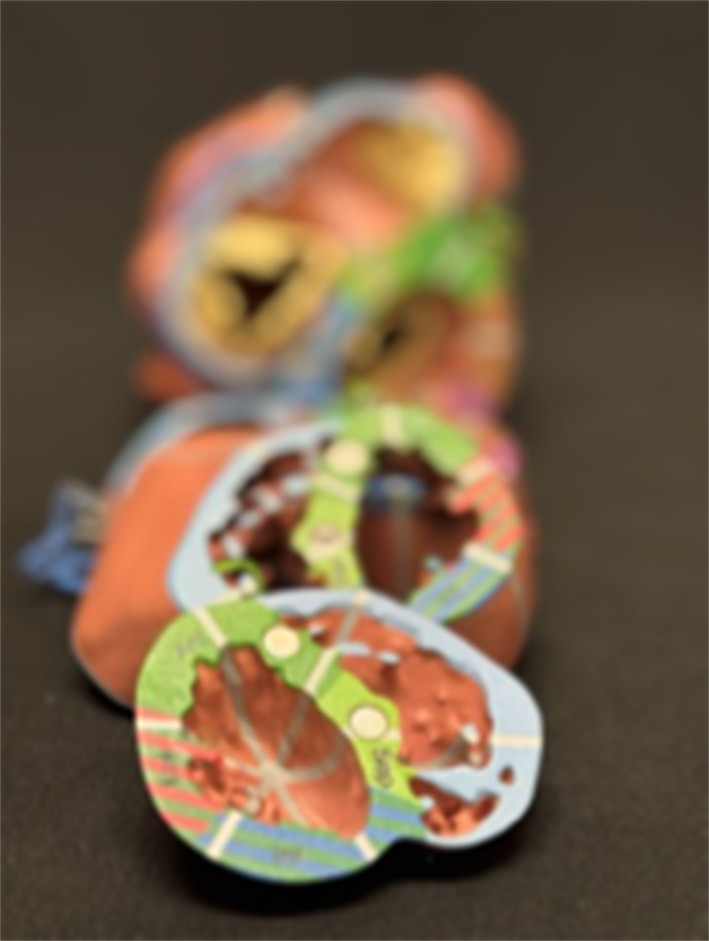
Magnetic 3D-printed cardiac model

Over the past three decades, medical education has increasingly embraced open-source learning tools that are regularly updated and shared among students. In parallel, the broader 3D printing community—often referred to as the ‘maker scene’—has thrived on principles of accessibility, diversity, and collaboration. While this open-source approach has fueled rapid innovation, its application in medicine requires careful consideration, particularly when developing patient-specific medical devices. However, for medical students, the focus remains on learning fundamental 3D printing skills, understanding anatomical structures, and exploring its role in clinical decision-making. For example, magnetic 3D-printed models of the heart (Figure 3) bring complex anatomy to life and allow students to tailor their learning experience to their unique needs. By aligning medical education with the collaborative nature of the 3D printing community, students gain access to valuable learning resources while maintaining responsible application of the technology.

These proposed curricula aim to equip students with essential skills in 3D printing technology, providing a blend of theoretical knowledge and practical application. This initiative not only meets the educational needs of students but also aligns with the future direction of medical practice, ensuring graduates are well prepared for the evolving landscape of healthcare technology. This project fills the initial, large unmet need for a blueprint so that medical schools can adopt and accelerate a 3D printing curriculum.

### Providing learners access to clinical data

To support hands-on learning, the readership will have access to a repository of 3D models and imaging data. The Supplemental Material includes several validated DICOM (.DCM) files and surface mesh files to support implementation [11]. This material is provided with permission for secondary use granted by the Institutional Human Research Committee (Institutional Review Board). Because these examples are not exhaustive, they should be used alongside other open anatomical databases. Medical schools may adapt them for case-based or problem-based learning, depending on their preferred curricular structure.

**Fig. 4:**
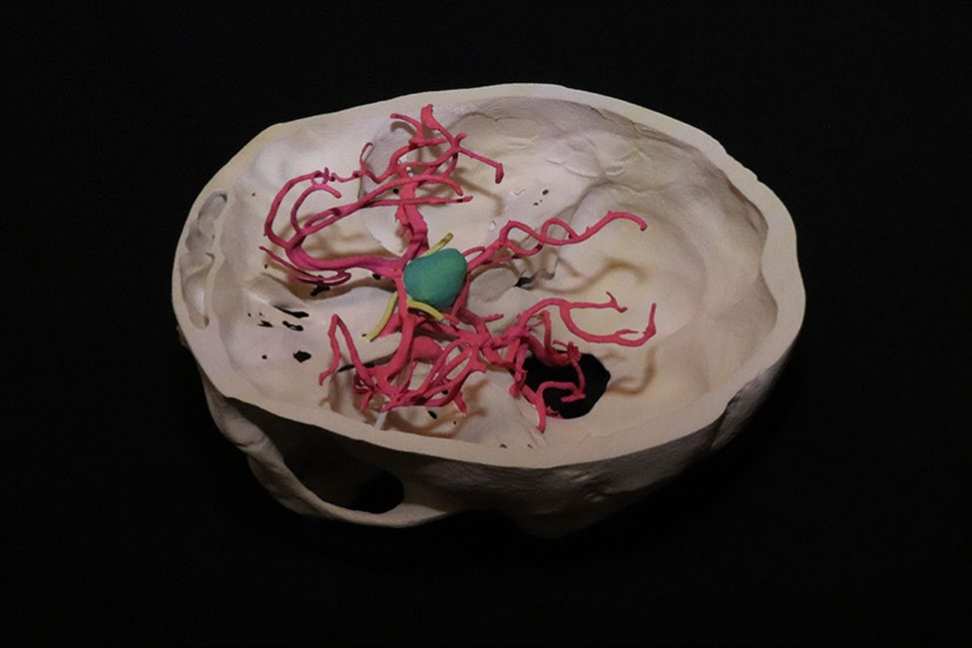
3D-printed Circle of Willis. 3D curricula would allow for students to explore complex structures such as the Circle of Willis, allowing for deeper understanding of concepts (e.g. arterial supply) and procedures (e.g. surgical approaches to resect the sellar mass in green)

By working with real medical imaging data, students gain practical experience in processing and printing models for educational and clinical applications. For example, a 3D-printed anatomic model of the skull base, Circle of Willis and arterial branches, and a mass (Fig. 4) enhances comprehension of the arterial system for anatomy as well as for understanding the strategies to resect a mass in the sella. This structured approach ensures that students are exposed to the same level of data quality used in clinical practice, bridging the gap between theoretical knowledge and real-world implementation.

### Developing engineering and faculty roles for program implementation

There are several 3D printing programs in medical education. However, to our knowledge, none of the curricula have been standardized. Regardless of how it is adopted, a medical school champion and a technical administrator/engineer is needed to ensure success. The engineering faculty can be drawn from a university-based school of engineering or can be hired by the medical school itself. Involvement of medical teaching faculty for each of the three curricula will depend on the initial interests of the program. At onset, we expect faculty to include radiology, surgery, and surgical subspecialties (e.g. oral and maxillofacial surgery, otolaryngology).

At the outset, the medical champion should be allocated no less than 0.2 FTE for the lab activities. The engineering champion is not required to have a medical background but instead must be familiar with the mechanics of 3D printing to help students troubleshoot, and to provide a liaison between the lab and the medical school physical plant. To ensure long-term sustainability, universities must allocate space, technical support, and funding to these programs as they expand.

The workload can become heavy for this engineer, necessitating up to one full FTE during the lab “busy” operations. For example, there may be several parts per day printed if 3D printing is located within a core anatomy curriculum. If medical students are participating in an elective, there will be “downtime” so that the person hired will have additional time to spend on other activities. For example, the engineer can be hired as 0.6 FTE in the 3D printing lab, and 0.4 elsewhere.

Regardless of the location of the lab (simulation center, medical library, or freestanding facility), the remaining time can be allocated to other activities as the program ramps up. As an example, when a program passes a specific threshold, additional human resources will be needed. In this regard, the lab is not different from other scalable resources, although the expertise (mechanical engineering) of the people required is unique.

### Teaching software and printing skills through guided instruction

Each curriculum will require hands-on instruction with students as they learn to navigate the software used for 3D printing. There are several commercial and free software packages for development of 3D prints from patient imaging; some commercial packages have student editions that can be used. Lessons should be conducted through a mix of virtual and in-person learning. Development of lecture material will depend on which curriculum is being pursued [2]. For example, anatomy-based integration would require development of projects/lectures complementary to students’ current organ-based learning. Alternatively, there are recorded tutorials available on YouTube that should be used as reference material [12]. In our case, we utilized both engineer-led instruction and recorded YouTube lectures to aid in segmentation and design. Students will advance their skills through independent practice with periodic feedback. In-person 3D printing and post processing should be led by 3D printing lab faculty instructors as troubleshooting is frequently needed during the printing process.

### Planning budget and resource requirements

Overhead: To be determined by medical school facilities.

Human Resources: 0.2–1.0 FTE engineer, matched to lab’s scope.

Hardware/Software: Matched to lab’s scope. Hardware to include 3D printers and computers for Computer Aided Design.

Post-Processing 3D Printed Parts: Matched to lab’s hardware.

Consumables: Dependent on lab hardware.

Research and Other Costs: Applications to human research committee (IRB), funding for registries and travel to present data.

## Supplementary Information

Below is the link to the electronic supplementary material.

Supplementary Material 1

## Data Availability

Not applicable.
